# Editorial: The pathogenesis and novel treatment options in AIBDs

**DOI:** 10.3389/fimmu.2026.1858274

**Published:** 2026-04-30

**Authors:** Sihan Liu, Hongjiang Qiao, Zhi Liu, Hui Fang, Pei Qiao, Yagang Zuo

**Affiliations:** 1 Department of Dermatology, State Key Laboratory of Complex Severe and Rare Diseases, Peking Union Medical College Hospital, Chinese Academy of Medical Sciences and Peking Union Medical College, Beijing, China; 2 Department of Dermatology, Xijing Hospital, Fourth Military Medical University, Xi’an, Shaanxi, China; 3 Department of Dermatology, School of Medicine, University of North Carolina at Chapel Hill, Chapel Hill, NC, United States

**Keywords:** autoimmune blistering diseases, BAFF, dupilumab, ITGB4, low-dose rituximab

Autoimmune blistering diseases (AIBDs) comprise a diverse group of disorders driven by pathogenic autoantibodies against structural proteins of the skin and mucous membranes, most notably bullous pemphigoid (BP) and pemphigus. Although therapeutic options have advanced considerably over the past two decades, challenges persist in optimizing treatment outcomes and unraveling the complex mechanisms underlying disease pathogenesis. This Research Topic brings together nine articles that collectively advance knowledge across these key areas.

## Therapeutic innovations: balancing efficacy and safety

Two systematic reviews and meta-analyses have individually evaluated the use of biologic therapies in treating AIBDs. Chen et al. assessed dupilumab in BP and reported a remarkable disease control rate of 95% and a complete response rate of 68%, with a favorable safety profile. These findings position dupilumab as a promising steroid-sparing option, particularly for patients with contraindications to conventional immunosuppression. Liu et al. examined low-dose rituximab in pemphigus and demonstrated comparable efficacy between low-dose and high-dose regimens across multiple outcomes, including complete remission and relapse rates. While no fatal events occurred, low-dose rituximab still requires vigilant monitoring. Beyond rituximab, other targeted B-cell therapies—such as ofatumumab, telitacicept, belimumab, and obinutuzumab—have been reported in several case reports for pemphigus treatment (1). Nonetheless, large-scale clinical studies are still lacking to further substantiate the efficacy of these biologic agents. The complexity of biologic therapy is further highlighted by Hu et al., who described a case of pemphigus erythematosus emerging during secukinumab treatment for psoriasis. This report serves as an important clinical reminder that cytokine-targeted therapies, while generally safe, may paradoxically trigger autoimmune responses in susceptible individuals through mechanisms involving immune dysregulation, autoantibody production, and genetic predisposition. In addition to secukinumab, etanercept, adalimumab, infliximab, and tocilizumab have also been identified as drugs associated with the risk of pemphigus (2, 3).

## Unraveling pathogenic mechanisms

Several studies have explored the underlying mechanisms of AIBDs, pointing to potential targets for future interventions. Hong et al. analyzed cytokine profiles in BP patients and found elevated serum levels of IL−17, IL−18, IL−22, and IL−25 compared to healthy controls, suggesting these cytokines could serve as useful biomarkers for monitoring disease activity. Furthermore, studies have shown that Th2 cells secrete IL-13 and interact with fibroblasts and dendritic cells via the IL-13–IL-13RA1 ligand–receptor pair, serving as a key pathogenic mechanism in BP (4). This finding reinforces the classification of BP as a type II inflammatory skin disease and paves the way for precision-targeted therapies.Li et al. reviewed the BAFF system across multiple autoimmune diseases, including BP and pemphigus vulgaris, and showed that increased BAFF levels correlate with autoantibody titers, disease activity, and relapse following B−cell depletion. Using lectin microarray assays, Miao et al. examined aberrant glycosylation patterns in BP, and notably, salivary glycosylation patterns were found to reflect disease severity, suggesting the potential for a non-invasive diagnostic tool that warrants further exploration.

## Diagnostic advances and rare phenotypes

Accurate and timely diagnosis is fundamental to optimal disease management. Zhang et al. evaluated a novel fully automated chemiluminescent immunoassay capable of quantifying four major autoantibodies from a single sample, demonstrating superior diagnostic performance to ELISA and good agreement with IIFT-BIOCHIP. Meanwhile, He et al. expanded the recognized clinical spectrum of junctional epidermolysis bullosa by reporting a patient with an ITGB4 mutation who presented with a previously undocumented bladder phenotype, highlighting the need for early urological evaluation in patients with unexplained urinary symptoms to help prevent chronic complications.

Collectively, these nine articles shed light on various aspects of AIBD research, spanning from bench to bedside. The therapeutic studies support the feasibility of de-escalation strategies for biologic agents. The mechanistic work points to novel biomarkers that could help guide personalized treatment. On the diagnostic front, new approaches offer the potential for simpler, faster, and more informative testing. As efforts continue to untangle the intricate interplay between innate and adaptive immunity in AIBDs, the findings presented here stand to inform both clinical practice and future research directions.
